# 982. Effect of Hepatic Impairment on the Safety and Pharmacokinetics of Rezafungin

**DOI:** 10.1093/ofid/ofab466.1176

**Published:** 2021-12-04

**Authors:** Jade Huguet, Voon Ong, Taylor Sandison, Rebeca M Melara, Thomas C Marbury, Alena Jandourek, Shawn Flanagan

**Affiliations:** 1 Altasciences, Montreal, Quebec, Canada; 2 Cidara Therapeutics, Inc., San Diego, California; 3 Orlando Clinical Research Center, Orlando, FL; 4 San Diego, California

## Abstract

**Background:**

Rezafungin (RZF) is a novel echinocandin antifungal being developed for treatment of candidemia and invasive candidiasis, and for prevention of invasive fungal diseases among immunosuppressed patients. In the Phase 2 and Phase 3 treatment trials of rezafungin compared with caspofungin (STRIVE [NCT02734862] and ReSTORE [NCT03667690], respectively), patients with severe hepatic impairment (HI) were not included due to lack of caspofungin data in this population. Rezafungin was previously evaluated in patients with moderate hepatic impairment. Here we report an open-label, single-dose study on rezafungin in patients with HI (Child-Pugh class C).

**Methods:**

To investigate the safety, tolerability, and pharmacokinetics (PK) of RZF in subjects with HI and healthy subjects (HS), 8 subjects with HI and 8 HS matched for age, sex, and body mass index (BMI) were enrolled and received a single 400-mg intravenous 1-hour infusion of RZF. Plasma PK sampling was performed at various time points through 336 hours postdose. RZF PK parameters were derived using non-compartmental analysis. Safety was assessed throughout the study.

**Results:**

The majority of the HI subjects were White (87.5%) and male (75%) while equal distribution between White and Black or African American was observed among HS (50%) and 75% were male. The mean age of HI subjects was 58 years (range, 41–68 years) and 56.6 years (range, 50–61 years) for the HS. Mean BMI was 29.7 kg/m^2^ (range, 24.5–34.3 kg/m^2^) for HI subjects and 29.7 kg/m^2^ (range, 25.4–34.2 kg/m^2^) for the HS. RZF exposure (C_max_ and AUC) in subjects with HI was ~30% lower than that in HS (Table 1), while half-life was generally similar (HI: 121 h, HS:124 h; Figure 1). Three HI subjects had one adverse event (AE) each (bronchitis, worsening hepatic encephalopathy, hyponatremia), all moderate in severity; one HS had 1 AE of infusion site infiltration mild in severity. No AEs were considered related to RZF, and all were resolved or resolving by the end of the study.

Table 1. Plasma Rezafungin PK Parameter Estimates in Subjects with Severe Hepatic Impairment or Normal Hepatic Function After a Single 400 mg IV Infusion of Rezafungin

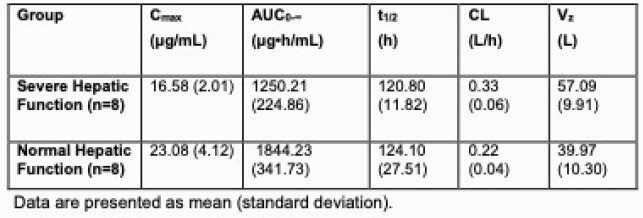

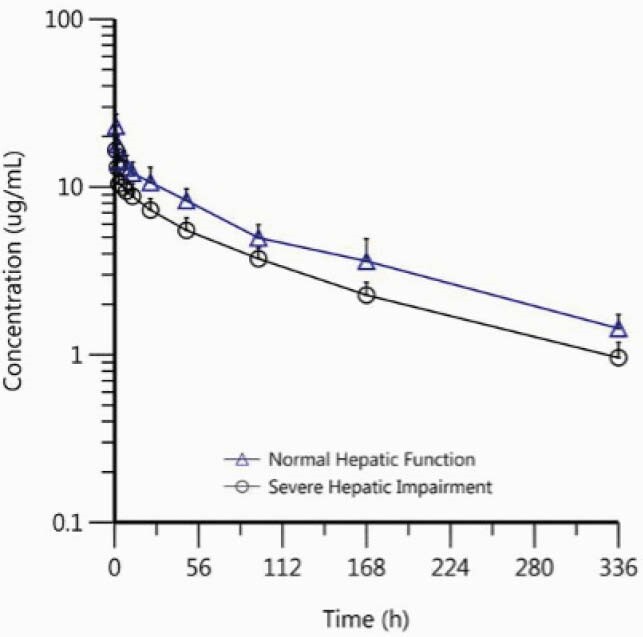

Figure 1. Mean (+SD) Plasma Rezafungin Concentration-Time Profiles in Subjects with Severe Hepatic Impairment or Normal Hepatic Function After a Single 400-mg IV Infusion of Rezafungin (Semi-Logarithmic Scale)

**Conclusion:**

RZF was well tolerated in HI subjects and showed modestly reduced exposure that was within the range observed in matched HS. These findings support no RZF dose adjustment in subjects with severe hepatic impairment.

**Disclosures:**

**Voon Ong, PhD**, **Cidara Therapeutics** (Employee, Shareholder) **Taylor Sandison, MD, MPH**, **Cidara Therapeutics** (Employee, Shareholder) **Rebeca M. Melara, M.S.**, **Altasciences (contract research organization**) (Employee) **Thomas C. Marbury, MD**, **Orlando Clinical Research Center** (Employee, Other Financial or Material Support, Equity owner of Orlando Clinical Research Center) **Alena Jandourek, MD**, **Cidara therapeutics** (Consultant) **Shawn Flanagan, PhD**, **Cidara Therapeutics** (Employee, Shareholder)

